# Research on Resource Recovery and Disposal of Copper-Containing Sludge

**DOI:** 10.3390/ma17112636

**Published:** 2024-05-29

**Authors:** Jinao Yu, Yongmin Zhou

**Affiliations:** College of Materials Science and Engineering, Nanjing Tech University, South Puzhu Road No. 30, Nanjing 211816, China; 202161203219@njtech.edu.cn

**Keywords:** copper-containing sludge, suspension state technique, decomposition characteristics under oxidising atmosphere, reduction characteristics

## Abstract

Copper-containing sludge is a common by-product of industrial activities, particularly electroplating and metal processing. This type of sludge contains high concentrations of heavy metals such as copper, which can pose a potential threat to the environment. Therefore, its treatment and disposal require special attention. Due to its efficient mass and heat transfer characteristics, the suspended state technology has shown significant potential for application in a number of key processes, including the drying, decomposition, and reduction of copper-containing sludge. This paper presents an in-depth analysis of the current status of the application of the suspended state technology in the treatment of copper-containing sludge. Based on this analysis, a device for the treatment of copper-containing sludge in the suspended state was designed, through which the characteristics of copper-containing sludge in the oxidative decomposition and reduction phases are investigated. The research objects were gas concentration, temperature, contact state, and particle size time. Orthogonal experiments were initially employed to investigate the relationship between the influencing factors and the conversion rate of copper oxides. This was followed by a single-factor influence study, which led to the determination of the optimal process parameters for the decomposition experiments of the Cu-containing sludge in an oxidizing atmosphere. The 100 μm Cu-containing sludge was reacted with 10% O_2_ gas at a flow rate of 1 m/s for 3 min under the condition of 900 °C. The process parameters were then determined as follows: The research objects were gas concentration, temperature, contact state, and particle size time. Orthogonal experiments were employed to investigate the relationship between the influencing factors and the copper conversion rate. This was followed by a single-factor influence study, which determined the optimal process parameters for the copper-containing sludge reduction experiments. The 200 μm copper-containing sludge was reacted for 5 min at a flow rate of 7% carbon monoxide at a flow rate of 1.5 m/s under the condition of 800 °C.

## 1. Introduction

Against the backdrop of the current environmental and energy crises, the resource utilisation of industrial by-products has become an important issue in scientific research and industry. Copper-containing sludge is a common solid waste and a common by-product of industrial wastewater treatment, especially in industries such as electroplating, semiconductor manufacturing, and mineral processing. This type of sludge is typically rich in heavy metals such as copper, and its treatment and disposal must be carefully managed to prevent environmental pollution and the waste of resources. Various recovery technologies for treating metals in copper-containing sludge have been developed, including acid leaching, ammonia leaching, microbiological methods, and thermal treatment. These technologies not only facilitate the recycling of resources but also reduce the environmental burden of waste disposal. In acid leaching [1,2], Li C et al. [3] achieved an effective technique for the extraction and recovery of multiple heavy metals from real electroplating sludge by ultrasonically enhanced two-stage acid leaching process. Deng et al. [4] leached copper from electroplating sludge containing a variety of metals such as Cu and Fe through a mixture of EDTA and citric acid, and the selective recovery of copper was achieved through electrodeposition using functionalised porous carbon (Ami-PC) electrode materials. Deposition, the selective recovery of copper, was achieved. Yuxin Z et al. [5] explored a new method for the stepwise recovery of iron, copper, zinc, and nickel from real electroplating sludge by coupled acid leaching and hot water extraction pathways. Ruijing S et al. [6] investigated the effect of leaching heavy metals from electroplating sludge by adding phosphate to a hydrochloric acid medium. In terms of ammonia leaching [7], the ammonia leaching process proposed by Xu W et al. [8] focused on the efficient and selective extraction and recovery of copper and nickel from electroplating sludge. Xiao Y [9] et al. proposed a combination of ammonia leaching and aluminium thermal reduction for the recovery of copper, chromium, and nickel from electroplating sludge. Ku Heesuk et al. [10] investigated an ammonia leaching method for the recovery of heavy metals from the cathode materials of used lithium-ion batteries using ammonia, ammonium thiosulphate, and ammonium carbonate. Chunhong Shi et al. [11] used a new method for the selective recovery of nickel from stainless steel pickling sludge (SSPS) using the NH3-(NH_4_)_2_CO_3_ ammonia leaching system. In terms of microbiological methods [12], the study conducted by Yang et al. [13] optimised the recovery of nickel from electroplating sludge by comparing three different bioleaching systems, especially using the sulphur–sulphate bacillus system. Nikfar et al. [14] succeeded in recovering nickel and chromium from chromium-enriched electroplating sludge for the first time by using the filter cultures of Aspergillus niger. Tian B et al. [15] explored a new method for the efficient recovery of nickel, copper, zinc, and chromium from electroplating sludge using an indirect bioleaching process and optimised the bioleaching process by response surface methodology. In terms of thermal treatment methods [16], Xu J et al. [17] proposed an innovative method to recover copper and arsenic from copper electrolysis sludge and copper waste by first performing the oxidative sintering of copper electrolysis sludge. Gao T r et al. [18] proposed a sulphide roasting–flotation–magnetic separation technique to effectively recover copper, nickel, zinc, and iron from electroplating sludge. Wang H Y et al. [19] proposed a new process incorporating a carbothermal reduction stage and a smelting stage for the effective recovery of nickel from nickel-containing electroplating sludge. Tang Z et al. [20] explored the recovery of iron, chromium, and nickel from pickling sludge using coal-based metallurgical reduction.

Suspended state technology [21] has shown significant potential for application in several key aspects of the decomposition and reduction of copper-containing sludge due to its efficient mass and heat transfer characteristics [22]. Currently, the application of a suspended state is mainly focused on improving the treatment efficiency and optimising the reaction kinetics [23], especially in promoting the efficient decomposition of organic matter in sludge and the effective reduction of oxides.

In terms of suspension decomposition, the study by Zhang H et al. [24] successfully activated low-quality gangue to prepare high-performance low-carbon cement through the suspension state calcination technology, demonstrating the high efficiency and potential commercial value of this technology in industrial-scale applications. Simeng C et al. [25] investigated the highly efficient decarbonisation and calcination process of coal-based kaolin by means of a suspended-bed reactor and thermal infrared analysis. A new kinetic model was developed to optimise the calcination parameters and high-quality calcined kaolin was successfully prepared. In terms of suspension reduction, Hanquan Z et al. [26] effectively converted manganese dioxide to manganese monoxide in soft manganese ores by an innovative suspension reduction roasting process, achieving high efficiency of manganese conversion and the selective removal of iron. Dong M et al. [27] proposed an innovative process to convert phosphogypsum to CaS and CaO in a fluidised bed using coal gasification fines as a reductant, achieving the high-efficiency resourcing of these industrial by-products and the reduction in environmental burden. Guifang Z et al. [28] improved the dephosphorisation process of ferrosilicon alloys by means of an electromagnetic levitation technique and a mixed reducing atmosphere of hydrogen and argon, demonstrating the significant advantages of this method in improving metal purity, preventing container contamination, and achieving rapid high-temperature melting.

This paper presents an in-depth analysis of the current status of the application of the suspended state technology in the treatment of copper-containing sludge. Based on this analysis, a set of devices for the treatment of copper-containing sludge in the suspended state is designed. The characteristics of copper-containing sludge in the drying, oxidative decomposition, and reduction phases are investigated through these devices.

We have chosen to recover copper from sludge containing copper, rather than other heavy metals, for the following reasons: Firstly, copper is the main heavy metal constituent of sludge and has a significant economic value, making its recovery more resource-efficient and economically beneficial. Secondly, copper has a high environmental impact, and its environmental and health risks can be significantly reduced through effective recycling. In addition, copper has a stable market demand and recycled copper is easy to sell, which increases the economic viability of the project. Finally, copper recycling faces fewer legal and administrative barriers, and is supported by policies and regulations, contributing to the smooth implementation of the project. Based on these combined factors, we believe that copper recycling is the most reasonable option.

## 2. Materials and Methods

### 2.1. Raw Materials

**Figure 1:**
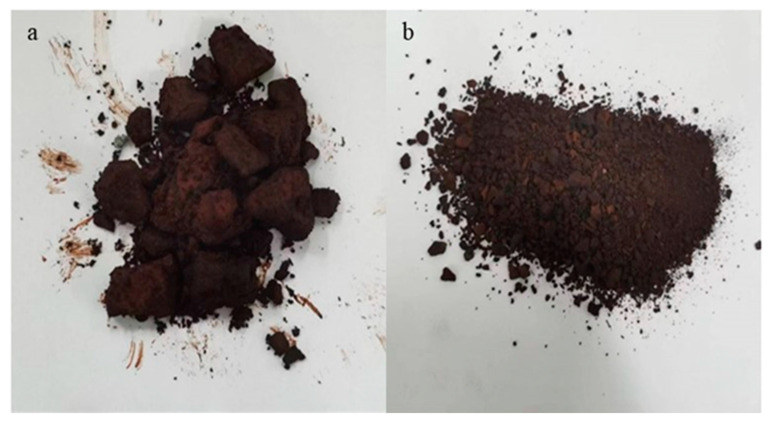
Copper-containing sludge before (**a**) and after drying (**b**).

The copper-containing sludge utilised in the experiment originated from an environmental protection technology company in Suqian, China, as illustrated in Figure 1. The raw material of the copper-containing sludge (a) exhibits a dark brown colour and a tightly bonded mud-like texture. The larger lumps have a tightly bonded mud-like texture, as shown in Figure 1. After drying, the volume of the copper-containing sludge shrinks drastically due to the high water content of the internal material, which is 68.6%.

### 2.2. Experimental Setup

**Figure 2:**
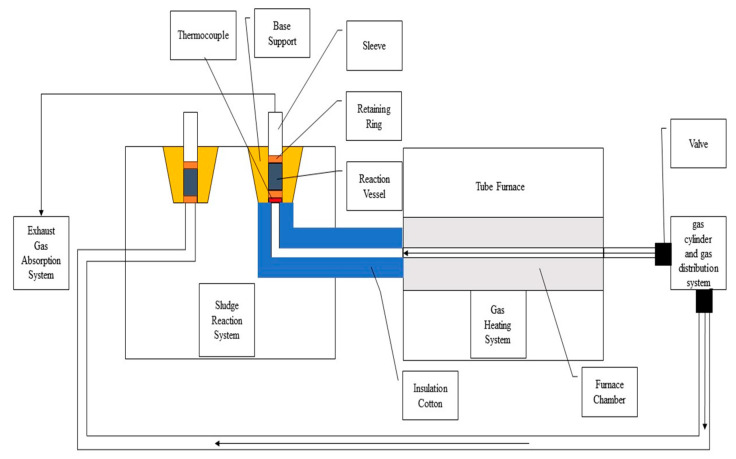
Schematic diagram of the experimental setup.

Figure 2 depicts the schematic diagram of the experimental setup, which comprises four systems: the gas cylinder and gas shunt system, the gas heating system, the sludge reaction system, and the tail gas absorption system. The atmosphere required for the experiment is provided by the gas cylinder. Initially, the gas flow is regulated by the gas shunt system, with the valve on the left side open and the valve on the lower side closed. Subsequently, the gas is heated as it passes through a stainless steel pipe within the chamber of the tube furnace. Subsequently, the heated gas enters the sludge reaction system via a stainless steel pipe insulated with heat-preserving cotton. The stainless steel pipe is connected to the bottom bracket, which serves to embed the casing equipped with thermocouples from the bottom to the top. The thermocouples provide the real-time measurements of the gas temperature. A ring is employed to maintain the position of the reaction pack, within which the sludge is placed and reacted. Subsequently, the exhaust gas flows through the end of the casing into the tail gas absorption system, where it is processed. To regulate the protective atmosphere, it is necessary to close the valve on the left side and open the valve on the lower side. Once the reaction has reached completion, the sludge is cooled and removed for testing. This occurs following the oxidative decomposition stage, which occurs at temperatures between 850 °C and 950 °C, and the reductive stage, which occurs at temperatures between 750 °C and 850 °C.

### 2.3. Experimental Methods

#### 2.3.1. Experimental Method for Decomposition of Sludge under Oxidizing Atmosphere

Weigh 1 g of completely dried sludge, noting that the initial sludge had a moisture content of 68.6%. Then, place the dried sludge into the reaction apparatus, and introduce the necessary oxidising atmosphere for the experiment to commence. Upon the completion of the reaction, the sludge must be removed from the apparatus. The reacted sludge should be dissolved in a solution comprising 1% sodium sulphite and 3% sulphuric acid. Once the solution has been suitably diluted, it should be analysed for copper content using inductively coupled plasma optical emission spectroscopy (ICP-OES, Agilent, in Santa Clara, CA, USA). The conversion rate of copper oxides is then calculated based on the following formula:

(1) $$\eta =\frac{C_{e}}{C}\times 100\%$$

where:

η—the conversion rate of copper oxides, %;

C_e_—the content of elemental copper in sludge after the dissolution of copper oxides in sludge under experimental conditions, %;

C—elemental copper content after complete calcination, %.

The decomposition reactions that may be involved in the oxidation experiments are as follows:

(2) $$Cu(OH{)}_{2}=CuO+H_{2}O$$

(3) $$CuSO_{4}=CuO+SO_{3}$$

(4) $$CuCO_{3}=CuO+CO_{2}$$

The oxidation reactions that may be involved in the oxidation experiments are as follows:

(5) $$2CuS+{3O}_{2}=2CuO+2SO_{2}$$

(6) $$Cu_{2}S+2O_{2}=2CuO+SO_{2}$$

(7) $$2Cu_{2}S+3O_{2}=2Cu_{2}O+2SO_{2}$$

#### 2.3.2. Experimental Methods for Sludge Reduction

Weigh 1 g of fully calcined sludge and transfer it to the reaction device. It is necessary to introduce the requisite reducing atmosphere for the experiment. Upon the completion of the experiment, the sludge must be extracted from the device. The reacted sludge should be dissolved in a 16% ferric chloride solution. Once the appropriate dilution has been achieved, the elemental copper content must be determined using inductively coupled plasma optical emission spectroscopy (ICP-OES). The conversion rate of copper monomer can then be calculated using the following formula:

(8) $$\eta =\frac{C_{e}}{C}\times 100\%$$

where

η—copper conversion rate, %;

C_e_—the content of copper and nickel elements in sludge after the dissolution of copper in sludge under experimental conditions,%;

C—the content of elemental copper after complete reduction,%.

Reduction reactions that may be involved in reduction experiments:

(9) $$CuO+CO=Cu+CO_{2}$$

(10) $${Cu}_{2}O+CO=2Cu+CO_{2}$$

The coper conversion rate: The term is employed to convey the impact of the conversion of copper oxides to copper monomers in fully calcined copper-containing sludge in copper-containing sludge reduction experiments. It represents the ratio of the content of copper monomers in copper-containing sludge that has undergone reduction under experimental conditions to the content of copper monomers in copper-containing sludge following complete reduction.

The objective of this study is to design experimental protocols for the oxidative decomposition and reduction phases of copper-containing sludge. A Taguchi L18 design was employed. The Taguchi L18 Orthogonal Arrays methodology represents an efficient approach to the design of experiments, offering a number of key benefits. This approach allows for a significant reduction in the number of experiments, thereby reducing both time and cost. Furthermore, the methodology provides a systematic framework for the rapid identification of critical factors, while also supporting the flexible treatment of multilevel factors. Furthermore, the methodology enhances product and process robustness and simplifies data analysis, thus increasing the reproducibility of experiments. Furthermore, the L18 array enables the analysis of finite interactions and multi-response optimisation, rendering it a valuable instrument in engineering and quality management when seeking optimal conditions, particularly in the context of multi-factor and multi-level challenges. These features render the Taguchi L18 not only resource-efficient but also more efficient and effective in research and development.

## 3. Results and Discussion

### 3.1. Copper-Containing Sludge Characteristics

**Figure 3:**
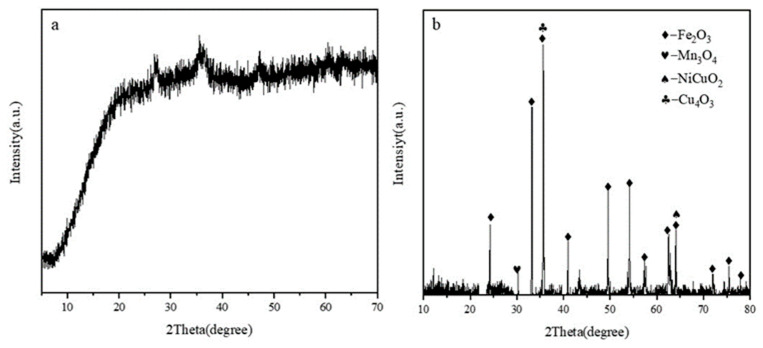
XRD image of raw copper-containing sludge (**a**) and XRD image of calcined copper-containing sludge (**b**).

An X-ray fluorescence analysis equipment (XRF) was employed to conduct a semi-quantitative analysis of the principal elements present in copper-containing sludge. The results of this analysis are presented in Table 1, which indicates that the content of Cu in the copper-containing sludge was 2.44%. This percentage is of high significance for recycling and application, with a content of Fe as high as 38.14%. The dark brown colour of the raw material containing copper-containing sludge may be due to the presence of Fe-containing compounds such as iron hydroxide and iron oxide. The physical phase of the copper-containing sludge was analysed by an X-ray diffraction (XRD, Bruker, in Karlsruhe, Germany) equipment. The original copper-containing sludge XRD diagram in Figure 3a shows that there are no obvious diffraction peaks, indicating that the metal in the original copper-containing sludge mainly exists in an amorphous form. The chemical deposition method employed in the treatment of the original copper-containing sludge may have resulted in the formation of metals in the form of amorphous hydroxides, which prevented the observation of characteristic peaks for iron, copper, and other elements in the original copper-containing sludge. As illustrated in Figure 3, the XRD diagram (b) of the calcined copper-containing sludge reveals that the predominant metals in the original copper-containing sludge existed in the form of metal oxides following calcination. The major compositions of the calcined copper-containing sludge were: Fe_2_O_3_, Mn_3_O_4_, NiCuO_2_, Cu_4_O_3,_ and so on, and the copper existed in the copper-containing sludge after calcination in the form of NiCuO_2_ and Cu_4_O_3_.

**Figure 4:**
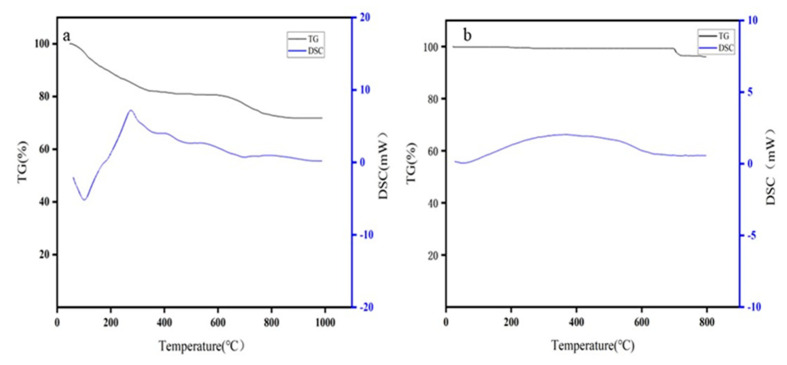
TG-DSC curves of copper-containing sludge under oxidizing atmosphere (**a**) and TG-DSC curves of copper-containing sludge under reducing atmosphere (**b**).

From Figure 4 of the TG-DSC curve of copper-containing sludge under an oxidizing atmosphere (a), it can be observed that the TG curve remains relatively unchanged at 850 °C, with a mass loss of 28.21%. Additionally, the DSC curve indicates that under an oxidizing atmosphere, the indeterminate hydroxides in the copper-containing sludge decomposed among themselves, resulting in a clear heat absorption peak. From Figure 4 TG-DSC curve of copper-containing sludge under reducing atmosphere (b), it can be seen that the TG curve tends to be unchanged at 750 °C, with a mass loss of 3.94%. From the DSC curve, it can be observed that the reduction reaction of NiCuO_2_ and Cu_4_O_3_ in the copper-containing sludge occurred under a reducing atmosphere, resulting in an evident exothermic peak.

### 3.2. Decomposition of Copper-Containing Sludge under Oxidising Atmosphere

#### 3.2.1. Orthogonal Experiment

A five-factor and three-level experimental scheme was selected for the orthogonal decomposition experiments on the copper-containing sludge under an oxidative atmosphere. Table 2 presents the factor table for the decomposition of the copper-containing sludge under an oxidative atmosphere. The atmosphere concentration of oxygen was varied between 4, 7, and 10%, while the temperature was set at 850, 900, and 950 °C. The contact state was varied across three levels (1, 1.5, and 2 m/s), the particle size was manipulated across three levels (100, 150, and 200 μm), and the time was varied across three levels (1, 3, and 5 min). Table 3 presents the orthogonal experimental table of L_18_(3^5^) and the corresponding results.

In Table 4, K1, K2, and K3 represent the sums of the copper oxide conversion rates for each factor at different levels, while k1, k2, and k3 represent the average copper oxide conversion rates at each level. The range analysis of copper oxide conversion rates in Table 4 shows that the order of influence of the five factors was as follows: temperature > gas concentration > particle size > time > contact state, with respective values of 4.46, 1.58, 0.66, 0.58, and 0.43. The range analysis indicates that in order to improve the conversion rate of copper oxide, the optimum process parameters should be a particle size of 100 μm for the copper-containing sludge oxidised at a temperature of 950 °C with a gas flow rate of 1 m/s and an oxygen concentration of 10% for 3 min, achieving a copper oxide conversion rate of 85.63%. Therefore, these parameters are selected for the subsequent single-factor influence experiments.

The objective of the oxidation reaction is to oxidise the copper compounds present in the copper-containing sludge, including copper sulphate, at elevated temperatures in order to convert them to copper oxides, thereby preparing the material for the subsequent reduction experiments. The final products are copper oxides, such as NiCuO_2_, Cu_4_O_3_, and so on.

#### 3.2.2. Single-Factor Experiments on the Decomposition of Copper-Containing Sludge under Oxidizing Atmosphere

**Figure 5:**
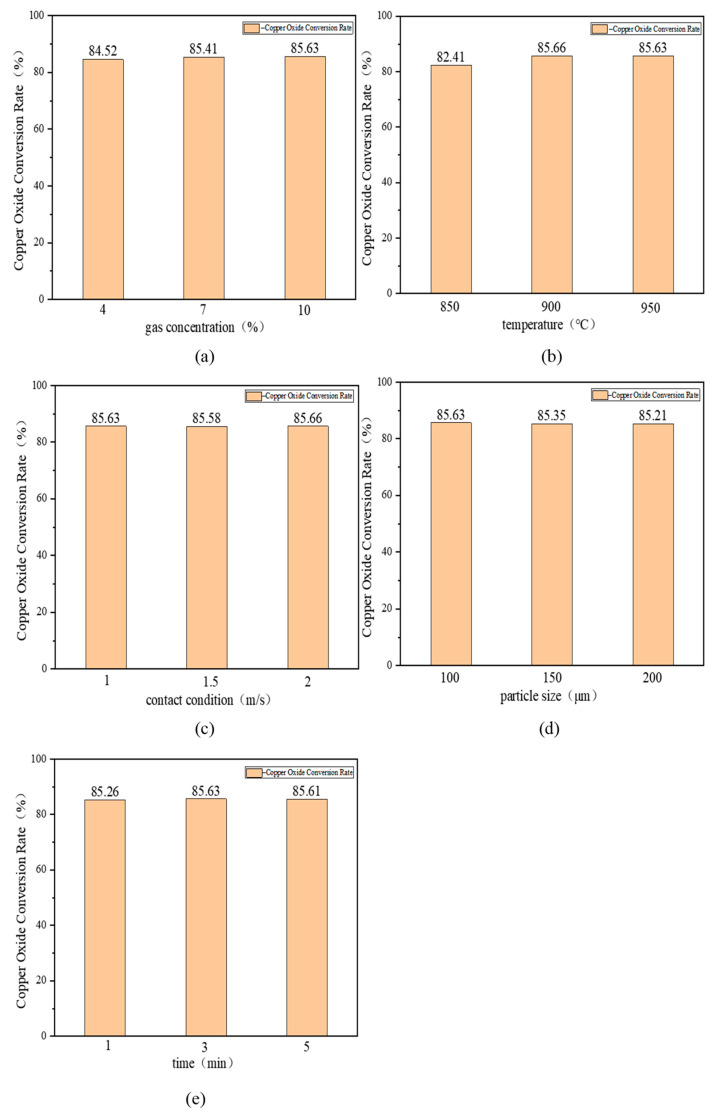
Variation in copper oxide conversion rate of copper-containing sludge under different factors: (**a**) gas concentration; (**b**) temperature; (**c**) contact condition; (**d**) particle size; (**e**) time.

Figure 5 illustrates the variation in the copper oxide conversion rate of copper-containing sludge in response to different factors. Figure 5a depicts the variation in the copper oxide conversion rate of copper-containing sludge under varying gas concentrations (4%, 7%, and 10%) at a constant temperature of 950 °C, a maintained contact state of 1 m/s, a fixed particle size of 100 μm, and a consistent reaction time of 3 min. The graphs demonstrate that the conversion rate of copper oxides increased as the atmospheric concentration increased. Specifically, the copper oxide conversion increased from 84.52% to 85.41% as the oxygen concentration increased from 4% to 7%, and further increased slightly to 85.63% as the oxygen concentration increased further to 10%. Oxygen was a necessary condition for the oxidation reaction, and the increase in oxygen concentration may have accelerated the rate of the oxidative decomposition of the copper-containing sludge, resulting in a greater quantity of copper oxides at the conclusion of the reaction. In the initial stages of the reaction, the oxidation reaction may be constrained by the rate of oxygen transfer due to the low oxygen concentration. As the oxygen concentration increases, the rate of the oxidation reaction increases, resulting in an increase in the yield of copper oxides. The reason for the small increase in the conversion of copper oxides at subsequent concentrations from 7% to 10% may be related to the fact that the rate of the reaction was limited by other factors (e.g., limits on elemental copper content) after a certain level of oxygen concentration had been reached. Furthermore, the oxidation process may approach equilibrium at higher oxygen concentrations, such that further increases in oxygen concentration do not have a significant effect on the production of copper oxides. In conclusion, the optimal atmospheric concentration should be 10%.

Figure 5b illustrates the variation in the conversion rate of copper oxides in copper-containing sludge at different temperatures (850 °C, 900 °C, and 950 °C). The experiment was conducted under the following conditions: a gas concentration of 10%, the contact state of 1 m/s, particle size of 100 μm, and reaction time of 3 min. From the figure, it can be observed that as the temperature increases, the conversion of copper oxides initially shows a significant increase, followed by a tendency to remain unchanged. This is due to the fact that temperature typically increases the reaction rate and increases the molecular motion and collision frequency of the reacting substances, thus promoting the chemical reaction. Specifically, as the temperature increases from 850 °C to 900 °C, the conversion rate of copper oxide increases from 82.41% to 85.66%. The increase in temperature typically accelerates the oxidation process, providing more kinetic energy to overcome the activation energy of the reaction. This may be the reason for the increase in the conversion rate of copper oxide at 900 °C compared to that at 850 °C. When the temperature continues to rise to 950 °C, the conversion rate of copper oxide becomes 85.63%. This change is relatively minor, potentially due to the copper-containing sludge at 900 °C. The oxidative decomposition of copper-containing sludge reaches equilibrium at this temperature, resulting in minimal further increase in the conversion rate of copper oxide at higher temperatures. In light of the aforementioned considerations, it was recommended that the temperature be set at 900 °C.

Figure 5c illustrates the variation in the conversion rate of copper oxide in copper-containing sludge under different contact states (1 m/s, 1.5 m/s, and 2 m/s) at a constant gas concentration of 10%, a temperature of 950 °C, a particle size of 100 μm, and a reaction time of 3 min. The figure illustrates that the conversion rate of copper oxides remained relatively constant with increasing contact conditions. At a contact state of 1 m/s, the copper oxide conversion rate was 85.63%; at a contact state of 1.5 m/s, the copper oxide conversion rate was 85.58%; and at a contact state of 2 m/s, the copper oxide conversion rate was 85.66%. This may indicate that the contact condition was not the primary factor controlling the rate of the oxidative decomposition reaction of the copper-containing sludge under the set conditions. Instead, the reaction may be controlled by the surface chemical reaction kinetics or diffusion processes within the sludge particles. This indicates that under the specified reaction conditions, the copper-containing sludge particles may have already reached a sufficient oxygen concentration for oxidation to reach its maximum rate. Consequently, increasing the contact state did not result in an increase in the conversion rate. Therefore, a contact state of 1 m/s was selected.

Figure 5d illustrates the variation in the conversion rate of copper oxide in the copper-containing sludge with different particle sizes (100 μm, 150 μm, and 200 μm) under the following specified conditions: a gas concentration of 10%, temperature of 950 °C, contact condition of 1 m/s, and reaction time of 3 min. From the figure, it can be observed that the conversion rate of copper oxides exhibited a slight increase with the reduction in particle size. The conversion of copper oxide increases from 85.21% to 85.35% for a reduction in particle size from 200 μm to 150 μm, and from 85.35% to 85.63% for a reduction in particle size from 150 μm to 100 μm. This may indicate that the diffusion of oxygen and the contact of reactants are sufficiently adequate within the experimentally set conditions to maintain an effective oxidative decomposition reaction even for larger particles (the particle size of the large particles is 200 μm). Overall, the particle size was chosen to be 100 μm.

Figure 5e illustrates the variation in the conversion rate of copper oxides in the copper-containing sludge at different times (1 min, 3 min, and 5 min) under a maintained gas concentration of 10%, a temperature of 950 °C, a contact condition of 1 m/s, and a particle size of 100 μm. From the figure, it can be seen that with the extension of time, the conversion of copper oxides initially increases, reaching a maximum of 85.63% at 3 min, before becoming constant. The copper oxide conversion increased from 85.26% to 85.63% when the time was prolonged from 1 min to 3 min, and the copper oxide conversion changed to 85.61% when the time was prolonged from 3 min to 5 min. This may indicate that the reaction of the oxidative decomposition of the copper-containing sludge rapidly reached a state of equilibrium or completion under high temperature and sufficient oxygen supply. The limited effect of increasing reaction time on the copper oxide conversion rate under these conditions suggests that the reaction may be rapid and that the rate of reaction at the initial stage determines the copper oxide conversion rate at the subsequent time points. All things considered, the time was chosen to be 3 min.

Accordingly, the optimal process parameters for the decomposition of the copper-containing sludge under an oxidising atmosphere are 10% gas concentration, 900 °C temperature, 1 m/s contact state, 100 μm particle size, and 3 min time.

### 3.3. Copper-Containing Sludge Reduction Experiment

#### 3.3.1. Orthogonal Experiment

A five-factor and three-level experimental scheme was selected for the orthogonal decomposition experiments on the copper-containing sludge under a reducing atmosphere. Table 5 presents the factor table for the reduction of the copper-containing sludge. The choices of carbon monoxide gas concentration were 1, 4, and 7%. The choices of temperature were 750, 800, and 850 °C. The contact state was varied between 1, 1.5, and 2 m/s, the particle size between 100, 150, and 200 μm, and the time between 1, 3, and 5 min. Table 6 presents the orthogonal experimental table of L_18_(3^5^) and the corresponding results.

In Table 7, K1, K2, and K3 are the sum of the copper conversion rate of copper-containing sludge corresponding to each level of each factor, and k1, k2, and k3 are the average values of copper conversion rate corresponding to each level, respectively. The results of the extreme variance analysis of the copper conversion rate in Table 7 show that the five influencing factors are in the order of temperature > gas concentration > time > particle size > contact state, which are 4.96, 2.57, 0.90, 0.39, and 0.36, respectively, and the results of the extreme variance analysis show that, from the point of view of improving the copper conversion rate, the optimal process parameters should be the copper-containing sludge with a particle size of 200 μm at a concentration of 7% carbon monoxide. The optimum process parameter were 200 μm particle size copper-containing sludge at a flow rate of 2 m/s and a temperature of 850 °C for 5 min at a concentration of 7% carbon monoxide, and the copper conversion rate was 83.75%, so these parameters were determined to be the parameters for the subsequent one-way influence experiments.

The objective of the reduction experiments was to reduce the copper oxides present in the fully calcined copper-containing sludge to copper monomers.

#### 3.3.2. Single-Factor Experiments on the Reduction of Copper-Containing Sludge

**Figure 6:**
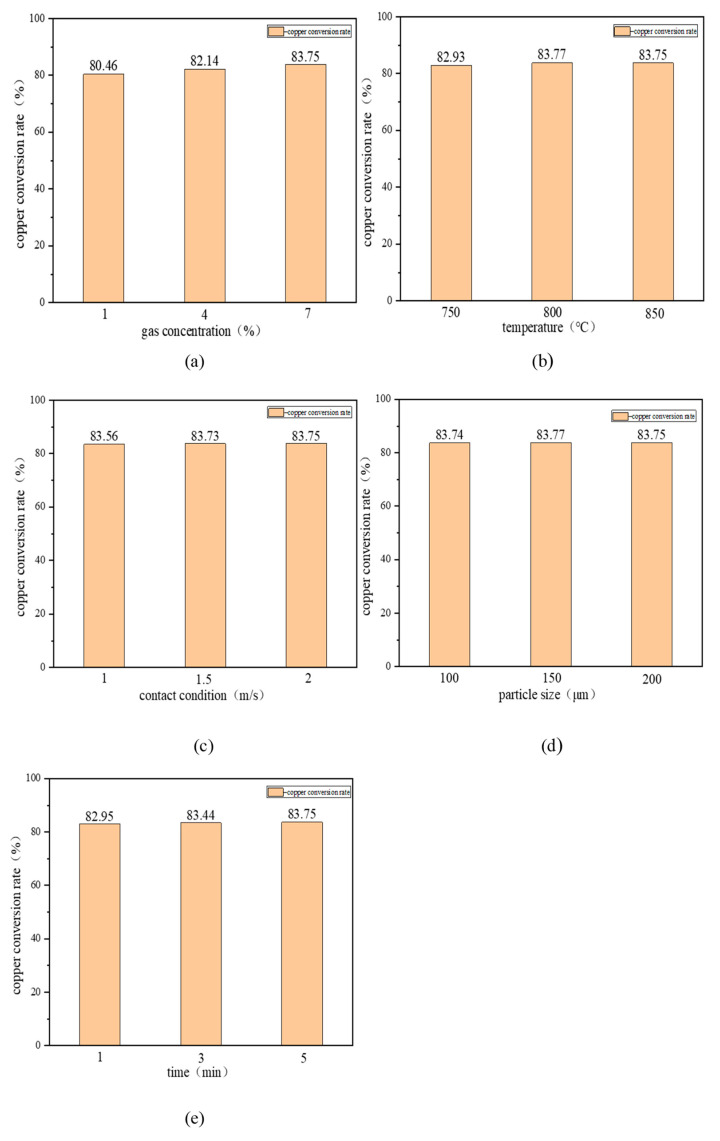
Variation in copper conversion rate of copper-containing sludge under different factors: (**a**) gas concentration; (**b**) temperature; (**c**) contact condition; (**d**) particle size; (**e**) time.

Figure 6 illustrates the variation in the conversion rate of the copper-containing sludge under different factors. Figure 6a depicts the variation in the copper conversion rate of the copper-containing sludge under different gas concentrations (1%, 4%, and 7%) at a constant temperature of 850 °C, a maintained contact state of 2 m/s, a fixed particle size of 200 μm, and a consistent reaction time of 5 min. As illustrated in the figure, the copper conversion rate exhibits a positive correlation with the gas concentration. The copper conversion rate demonstrates an upward trend, increasing from 80.46% to 82.14% when the gas concentration was elevated from 1% to 4%, and further increasing to 83.75% when the gas concentration was increased from 4% to 7%. Carbon monoxide, as a reducing agent, can effectively react with copper oxides and promote the reduction of copper when sufficient heat is provided. The increase in concentration means that the partial pressure of carbon monoxide in the reaction system is elevated, which provides more reducing conditions for copper oxides, which may lead to the forward progress of the copper reduction reaction, thus improving the copper conversion rate. Therefore, it can be concluded that the optimal gas concentration should be 7% (the chemical composition of the used gas is CO and N_2_).

Figure 6b illustrates the variation in the copper conversion of copper-containing sludge at different temperatures (750 °C, 800 °C, and 850 °C) under the following specified conditions: a gas concentration of 7%, contact condition of 2 m/s, particle size of 200 μm, and reaction time of 5 min. From the figure, it can be observed that with an increase in temperature, there is a notable initial rise in copper conversion, which then stabilises. From the figure, it can be seen that as the temperature increased from 750 °C to 800 °C, the copper conversion rate increased significantly from 82.93% to 83.77%. This may be due to the fact that at 750 °C, the copper-containing sludge had just reached the critical point of the activation energy. Further increases in temperature resulted in a significant increase in the rate of the reaction. In the 750 °C to 800 °C interval, the copper-containing sludge may have begun the rapid reduction in the starting point. The interval between 750 and 800 degrees Celsius may just cross the starting point of the rapid reduction of copper-containing sludge. As the temperature rises from 800 °C to 850 °C, the copper conversion rate undergoes a slight decline from 83.77% to 83.75%. This may be attributed to the fact that the reduction reaction has reached its maximum rate, resulting in a stabilisation of the conversion rate even as the temperature continues to increase.

Figure 6c illustrates the variation in the copper conversion of the copper-containing sludge under different contact states (1 m/s, 1.5 m/s, and 2 m/s) at a maintained gas concentration of 7%, a temperature of 850 °C, a particle size of 200 μm, and a reaction time of 5 min. As illustrated in the figure, the copper conversion initially exhibits a slight increase with an increase in contact state, followed by a tendency towards a constant value. From the graph, it can be seen that the copper conversion rate increases slightly from 83.56% to 83.73% when the contact state was raised from 1 m/s to 1.5 m/s, and becomes 83.75% when the contact state was raised from 1.5 m/s to 2 m/s, essentially converging to the same level. The increase in the contact state should theoretically result in an enhanced frequency of gas–solid contact, thereby enhancing the rate of the reduction reaction. This explains the slight increase in the conversion rate from 1 m/s to 1.5 m/s. However, when the contact rate was increased from 1.5 m/s to 2 m/s, the conversion rate remained almost constant. This suggests that the reaction may already be close to equilibrium at 1.5 m/s, and that a further increase in the contact rate may not have a significant effect on the conversion rate. Taken together, the contact state was chosen to be 1.5 m/s.

Figure 6d illustrates the variation in the copper conversion rate of the copper-containing sludge with different particle sizes (100 μm, 150 μm, and 200 μm) under the condition of maintaining a gas concentration of 7%, a temperature of 850 °C, a contact condition of 2 m/s, and a reaction time of 5 min. As illustrated in the figure, there is no discernible trend in the conversion rate of copper with an increase in particle size. Specifically, the copper conversion rate was 83.74% at a particle size of 100 μm, 83.77% at a particle size of 150 μm, and 83.75% at a particle size of 200 μm. This indicates that the impact of particle size on the conversion rate is minimal under these reaction conditions. This is likely due to the high temperature and sufficient reaction time, which enables the effective reduction of copper-containing sludge even with a large particle size. The particle size was selected to be 200 μm.

Figure 6e illustrates the variation in the copper conversion rate of copper-containing sludge at different times (1 min, 3 min, and 5 min) under the following specified conditions: a gas concentration of 7%, temperature of 850 °C, contact condition of 2 m/s, and particle size of 200 μm. As shown in the figure, the copper conversion rate exhibited a gradual increase with the extension of time. From the graph, it can be observed that the copper conversion rate increased from 82.95% to 83.44% when the time was extended from 1 min to 3 min, and from 83.44% to 83.75% when the time was extended from 3 min to 5 min. With the extension of time, the contact between the carbon monoxide and copper oxides in the copper-containing sludge became more adequate, allowing more copper oxides to have the opportunity to be reduced. This reflects the fact that at high temperatures, sufficient contact time is essential for the complete reduction of copper oxides. Although the reaction proceeded rapidly in the initial stage, more copper oxides were reduced with increasing time, leading to a gradual increase in the conversion rate. This phenomenon indicates that although the reaction proceeds rapidly during the initial stage, it does not reach equilibrium rapidly; rather, the system gradually moves towards equilibrium with increased time, increasing the overall copper conversion rate. Therefore, the optimal time selection should be 5 min.

Accordingly, the parameters of the copper-containing sludge reduction process should be selected as follows: a gas concentration of 7%, temperature of 800 °C, contact condition of 1.5 m/s, particle size of 200 μm, and time of 5 min.

## 4. Conclusions

In this study, a copper-containing sludge treatment unit was fabricated by studying the current status of copper-containing sludge treatment and combining it with the suspended state technology. The copper-containing sludge treatment process was optimised and determined by studying the characteristics of copper-containing sludge in the decomposition phase and the reduction phase under the oxidising atmosphere. The following conclusions were drawn:

(1) In the decomposition experiments of the copper-containing sludge under an oxidising atmosphere, the factors influencing the conversion rate of copper oxide were ranked as follows: temperature > gas concentration > particle size > time > contact state. The highest conversion rate of copper oxide in the copper-containing sludge was achieved under the following process parameters: a gas concentration of 10%, temperature of 950 °C, contact state of 1 m/s, particle size of 100 μm, and a duration of 3 min. Based on these findings, single-factor experiments were conducted within the selected ranges of each influencing factor. By analysing the patterns of copper oxide conversion rate under various conditions, the optimal process parameters for the decomposition of the copper-containing sludge in an oxidising atmosphere were determined to be a gas concentration of 10%, temperature of 900 °C, the contact state of 1 m/s, particle size of 100 μm, and a duration of 3 min. The copper oxide conversion rate was 85.66%.
(2) In the reduction experiments of the copper-containing sludge, the factors influencing the conversion rate of copper were ranked in the following order: temperature > gas concentration > time > particle size > contact state. The highest copper conversion rate was achieved under the following process parameters: a gas concentration of 7%, temperature of 850 °C, contact state of 2 m/s, particle size of 200 μm, and a duration of 5 min. Based on these findings, single-factor experiments were conducted within the selected ranges of each influencing factor. The patterns of copper conversion rate under different conditions were studied in order to determine the optimal process parameters for the reduction of copper-containing sludge. These were found to be a gas concentration of 7%, temperature of 800 °C, contact state of 1.5 m/s, particle size of 200 μm, and a duration of 5 min. The copper conversion rate was 83.75%.

The copper-containing sludge treatment device and the experimental method designed in this study demonstrated superior treatment efficiency compared with previous studies. This is a significant advantage over the treatment methods that require several minutes. The refined control system and optimised operating parameters employed in this study not only enhance the copper recovery rate but also reduce energy consumption and effectively mitigate energy waste. These innovations are directly supportive of the dual-carbon goal of achieving carbon peak and carbon neutrality. They provide an energy-efficient and effective solution for the treatment of copper-containing sludge, thereby promoting resource recycling and environmental sustainability.

The experimental setup employed in this study should be translated to real industrial production in the future. This will require the optimisation of parameters and the conduction of scale-up studies to ensure the effectiveness and stability of the technology on an industrial scale. Future studies should include a comprehensive evaluation of economic benefits, scale-up trials to ascertain the viability of the technology, and performance stability tests for long-term operation. Furthermore, it is of paramount importance to consider the environmental impact, compliance with regulations and safety standards, and the integration of the technology with existing industrial processes in order to successfully transition from laboratory to industrial applications. These measures will assist in the comprehensive assessment of the potential for the industrial applications of the technology and facilitate its commercialisation and continuous improvement.

## Figures and Tables

**Table 1:** Main elemental composition of copper sludge.

Chemical Composition	Fe	S	Cu	Ni	P	Ti	Mg	Na	Mn
Content/%	38.14	2.92	2.44	1.80	0.877	0.561	0.360	0.290	0.228

**Table 2:** Factor table for decomposition of copper-containing sludge under oxidising atmosphere.

Serial Number	Gas Concentration (%)	Temperature (°C)	Contact Condition (m/s)	Particle Size (μm)	Time (min)
1	4	850	1	100	1
2	7	900	1.5	150	3
3	10	950	2	200	5

**Table 3:** Five-factor three-level orthogonal table for decomposition of copper-containing sludge under oxidising atmosphere.

Serial Number	Factors					Copper Oxide Conversion Rate (%)
	Gas Concentration (%)	Temperature (°C)	Contact Condition (m/s)	Particle Size (μm)	Time (min)	
1	1	1	1	1	1	78.14
2	1	1	2	2	3	79.21
3	1	2	1	3	3	81.83
4	1	2	3	1	2	82.94
5	1	3	2	3	2	84.15
6	1	3	3	2	1	83.21
7	2	1	1	3	2	79.41
8	2	1	3	1	3	81.42
9	2	2	2	2	2	82.75
10	2	2	3	3	1	83.12
11	2	3	1	2	3	84.86
12	2	3	2	1	1	84.36
13	3	1	2	3	1	80.23
14	3	1	3	2	2	81.15
15	3	2	1	2	1	83.52
16	3	2	2	1	3	84.32
17	3	3	1	1	2	85.63
18	3	3	3	3	3	84.13

**Table 4:** Range analysis table for copper oxide conversion rate of copper sludge under oxidative atmosphere.

Serial Number	Factors				
	Gas Concentration (%)	Temperature (°C)	Contact Condition (m/s)	Particle Size (μm)	Time (min)
K_1_	489.48	479.56	493.39	496.81	492.58
K_2_	495.92	498.48	495.02	494.70	496.03
K_3_	498.98	506.34	495.97	492.87	495.77
k_1_	81.58	79.93	82.23	82.80	82.10
k_2_	82.65	83.08	82.50	82.45	82.67
k_3_	83.16	84.39	82.66	82.15	82.63
Range(R)	1.58	4.46	0.43	0.66	0.58

**Table 5:** Factor table for reduction of copper-containing sludge.

Serial Number	Gas Concentration (%)	Temperature (°C)	Contact Condition (m/s)	Particle Size (μm)	Time (min)
1	1	750	1	100	1
2	4	800	2	150	3
3	7	850	3	200	5

**Table 6:** Reduction of copper-containing sludge in five-factor three-level orthogonal table.

Serial Number	Factors					Copper Conversion Rate (%)
	Gas Concentration (%)	Temperature (°C)	Contact Condition (m/s)	Particle Size (μm)	Time (min)	
1	1	1	1	1	1	75.58
2	1	1	2	2	3	76.73
3	1	2	1	3	3	78.53
4	1	2	3	1	2	79.42
5	1	3	2	3	2	80.84
6	1	3	3	2	1	80.81
7	2	1	1	3	2	76.85
8	2	1	3	1	3	77.84
9	2	2	2	2	2	79.46
10	2	2	3	3	1	80.24
11	2	3	1	2	3	82.79
12	2	3	2	1	1	81.21
13	3	1	2	3	1	77.54
14	3	1	3	2	2	78.72
15	3	2	1	2	1	81.25
16	3	2	2	1	3	82.41
17	3	3	1	1	2	83.64
18	3	3	3	3	3	83.75

**Table 7:** Range analysis table for copper conversion rate in the reduction of the copper-containing sludge.

Serial Number	Factors				
	Gas Concentration (%)	Temperature (°C)	Contact Condition (m/s)	Particle Size (μm)	Time (min)
K_1_	471.91	463.26	478.64	480.10	476.63
K_2_	478.39	481.31	478.19	479.76	478.93
K_3_	487.31	493.04	480.78	477.75	482.05
k_1_	78.65	77.21	79.77	80.02	79.44
k_2_	79.73	80.22	79.70	79.96	79.82
k_3_	81.22	82.17	80.13	79.63	80.34
Range(R)	2.57	4.96	0.36	0.39	0.90

## Data Availability

The original contributions presented in the study are included in the article, further inquiries can be directed to the corresponding author.
